# Association between a miRNA-146a polymorphism and susceptibility to head and neck squamous cell carcinoma in Chinese patients: A meta-analysis of 8 case–control studies

**DOI:** 10.1371/journal.pone.0186609

**Published:** 2017-10-19

**Authors:** Silin Zhang, Fangling Hu, Hongxing Liang, Yuanzhou Liu, Jianqiang Yang, Wensheng Zhou

**Affiliations:** 1 Department of Otolaryngology, Shenzhen Hospital of Southern Medical University, Shenzhen, China; 2 Department of Otolaryngology, The First Affiliated Hospital of Nanchang University, Nanchang, China; Duke Cancer Institute, UNITED STATES

## Abstract

A closer association has been found between the microRNA-146a *rs2910164* polymorphism and the risk of head and neck carcinoma in some molecular epidemiological studies. Recently two meta-analyses were performed to explore the relationship between miRNA-146a polymorphisms and the susceptibility of squamous cell carcinoma of the head and neck (SCCHN); however, they yielded conflicting results in susceptibility regarding ethnic variations. Hence, the present study was performed to explain the relationship between the miRNA-146a *rs2910164* polymorphism and the risk of SCCHN development of Chinese patients. We retrieved databases and screened eligible papers up to March 10, 2017 and then we extracted the essential data. The subgroup analyses were also performed based on the tumor site, region, and genotyping means. Crude odds ratios (OR) at 95% confidence intervals (CI) were chosen to describe the strength of the association. As a result, 6 publications were included in our study which involved 8 independent case-control studies. A significant association was found between miR-146a *rs2910164* polymorphisms and the risk of SCCHN in Chinese patients according to the overall data [CC+CG vs. GG: OR = 1.13; 95%CI = 1.00–1.29; CC vs. GG: OR = 1.19; 95%CI = 1.03–1.38]. According to the subgroup analysis based on tumor site, the risk of cancer was significantly increased among laryngeal cancer (dominant model: OR = 1.76, 95%CI = 1.26~2.46, P = 0.001; homozygote model: OR = 1.83, 95%CI = 1.25~2.67, P = 0.002) and nasopharyngeal carcinoma (homozygote model: OR = 1.41, 95%CI = 1.05~1.90, P = 0.022). In summary, variant alleles of miR-146a *rs2910164* alleles may have an association with the increased risk of SCCHN in Chinese patients, and these associations differed based on tumor site. Further studies including a larger sample size will be necessary to clarify these results.

## Introduction

Squamous cell carcinoma of the head and neck (SCCHN) is the sixth common cancers [1]; approximately 53640 new cases and 11520 deaths were reported in 2013 in the United States[2]. Human papillomavirus (*HPV*) is etiologically responsible for a distinct subset of SCCHN. The incidence of *HPV*-associated squamous cell carcinoma is increasing now even though the incidence of *HPV*-negative SCC may be increasing in some geographical areas, while in general there is a constant decline [3, 4]. There are regional differences in the incidence of SCCHN, for which the epicenter includes southeast Asia, central Europe, eastern Europe, Latin America, and it was the most common tumor in India[5]. The effects of advanced SCCHN treatment are poor, which resulted in severe disability, poor survival rates and reduced life quality of patients.

Cancer is caused by unregulated gene expression related to environmental factors and the susceptibility of individuals`genetic. The etiology of SSCHN was associated with *HPV*, tobacco, alcohol, and some molecular drivers. Recently, studies elucidated a new mechanism of microRNA (miRNAs) mediated transcriptional regulation. MiRNAs are small fragment RNAs and are composed by approximately 20–22 nucleotides, which can negatively regulate the efficiency and stability of mRNAs translational by targeting specific mRNAs [6]. It has been demonstrated the expression of roughly 10~30% of all human genes can be regulated by mature miRNAs[7].

Aberrant expression of miRNA-146a is related to many cancers[8]. Meanwhile, more and more published papers has revealed that the *rs2910164 G>C* polymorphism of miRNA-146a was associated with a number of carcinomas[9] and there is an ethnicity-dependent relationship between the risk of cancer and the *rs2910164* polymorphism[10]. In recent years, four meta-analyses have studied the associations between miRNA-146a polymorphisms and the risk of SCCHN [11–14], but the results were conflict. Considering that the incidence of SSCHN differed by ethnicity and region and polymorphisms are associated with specific environments[15, 16], we performed a meta-analysis to study the association between the *rs2910164* polymorphism of miRNA-146a and SCCHN risk in Chinese patients.

## Materials and methods

### Publication search

To search all potentially eligible studies focus on the association of miRNA polymorphisms and the risk of SCCHN, we conducted a systematic search for all papers published up to March 10, 2017 on the PubMed, EMBASE, Web of Science, Chinese Nation Knowledge Infrastructure (CNKI), and WanFang databases. The search terms we used in this study were “miRNA-146a”, “hand and neck cancer”, “nasopharyngeal cancer”, “laryngeal cancer”, and “mouth neoplasm”. We also screened the references of the retrieved articles and review articles. The inclusion criteria of eligible studies were as follows: 1) full-text study, 2) study the relationship between miRNA polymorphisms and the risk of cancer, 3) independent case-control design, and 4) sufficient data to estimate a P-value and the odds ratio (OR) with 95% confidence interval (CI). Studies were considered as two or more independent studies if they had two or more case-control groups.

### Data extraction

The published papers which met the inclusion criteria were reviewed and extracted information independently by two investigators. The two investigators reached an agreement in the case of a conflict views by discussion. Each publication was sought for the following information: the surname of first author, the publication year, tumor site, region of organization, source of control groups, genotyping means, and the numbers of cases and controls for each genotype. The Newcastle-Ottawa Scale (NOS) was chosen to assess the methodological quality of case-control studies[17].

### Statistical analysis

To investigate the departure of the miRNA polymorphisms frequencies from expectation for each study under the Hardy-Weinberg equilibrium (HWE), the goodness-of-fit test (chi-square or Fisher exact test) in controls was used.

According to the methods published by Woolf et al.[18], the strength of association was assessed between miRNA polymorphisms and the risk of cancer by crude OR corresponding to 95% CI. Z-test was performed to determine the significance of the pooled OR, and a P-value of < 0.05 was considered statistically significant. The study was performed in allelic contrast (C vs. G), homozygote comparisons (CC vs. GG), heterozygote comparisons (GC vs. GG), dominant models (CC+GC vs. GG), recessive models (CC vs. GC+GG), and additive models (GG+CC vs GC) respectively to determine the relationship of genetic variants and the risk of cancer. According to tumor site (nasopharyngeal, oral, and laryngeal), region (Hong Kong, Taiwan, and Inland), and genotyping methods (Taqman, PCR, PCR-RFLP, and Illumina), we performed the subgroup analyses.

We conducted the analyses of between-study heterogeneity by a chi-squared-based Q-statistic test. If the P-value for the Q-test was >0.05, *I*^2^ was <50%, indicating the absence of heterogeneity, which yielded wide CIs, the Mantel-Haenszel method was chosen to calculate pooled OR in a fixed-effect model[19]; otherwise, the DerSimonian and Laird method was chosen to calculate pooled OR via a random-effect model [20].

The results stability and reliability was evaluated using sensitivity analysis. One study from the database was deleted each time and the analyses were repeated. We considered overall results of were stable if the data showed that the overall results were not statistically changed in the overall analysis.

Publication bias was examined visually from funnel plots, An evident publication bias might exist if the plot was asymmetrical. Egger`s linear regression test was used to evaluate the symmetry of the funnel plot in order to minimize the subjective influence of the visual inspection assessment [21]. If the funnel plot was asymmetrical or the P-value of the Egger`s test was <0.05, then that was considered an indication of potential publication bias. All statistical analyses were carried out using STATA 14.0 software (Stata Corporation, TX, USA).

## Result

### Study identification

**Fig 1:**
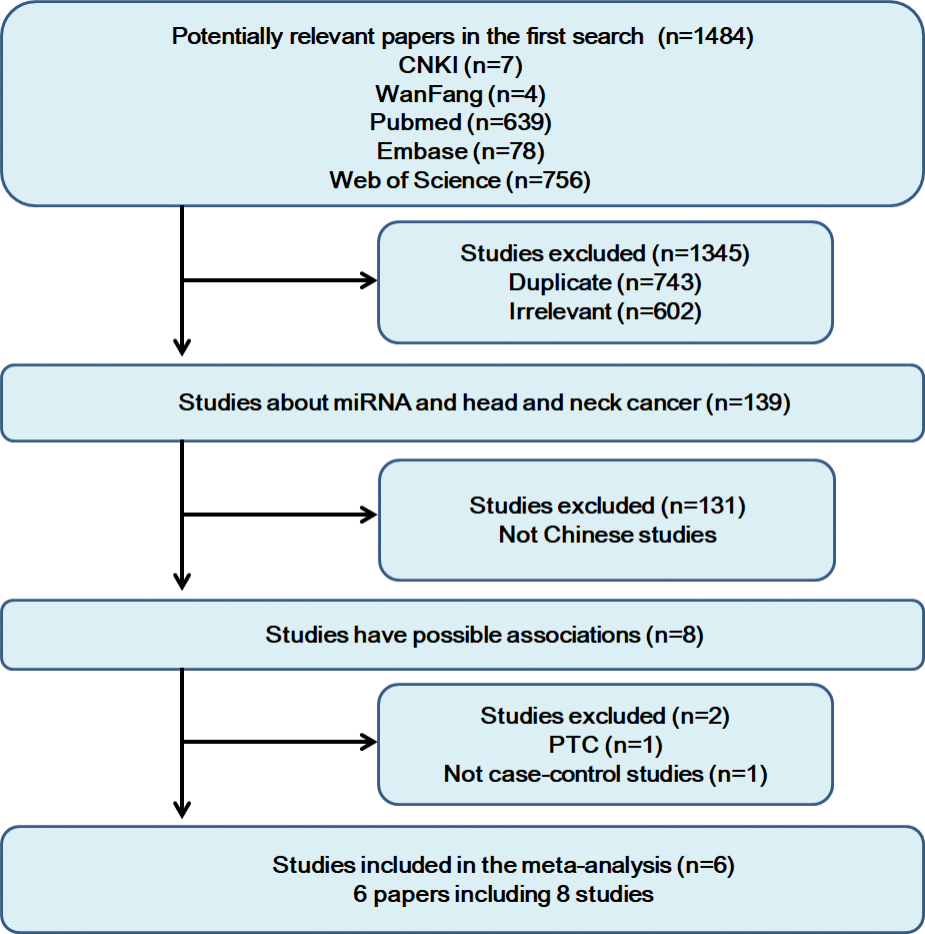
The flow diagram of included/excluded studies.

According to our search strategy, 1484 articles were initially screened, which included 639 articles from PubMed, 78 articles from Embase, 756 articles from Web of Science, 7 articles from CNKI, and 4 articles from WanFang. After removing duplicates and irrelevant studies, there were 139 articles left. Non-Chinese studies were excluded through author addresses and abstracts, which left 8 Chinese studies, including one non-case-control study[22] and one paper studied the association between the miR-146a and papillary thyroid carcinoma (PTC)[23]. Finally, 6 studies[24–29] discussed the association of miRNA-146a polymorphisms and the risk of SSCHN. Notably, the present meta-analysis included 6 papers but contained 8 case-control studies because there were two papers, by Chen et al. and Lung et al. respectively, included four independent studies (Fig 1).

Of the six papers, one article was a study for laryngeal cancer, two articles were studies for oral cancer, two articles were studies for nasopharyngeal carcinoma, and one article was a mixed study; three of them were from the mainland, two from Taiwan, and one from Hong Kong. In terms of genotyping methods, 2 articles used Taqman, 2 articles PCR-RFLP, 1 article PCR, and 1 article Illumina. For the miR-146a *rs2910164* polymorphism, our study included 2485 cases and 11034 controls. All NOS scores were greater than 5 stars (NOS scores greater than 5 stars define a good article). The genotypic distribution of most of the studied SNPs in controls agreed with HWE (P>0.05). More detail can be found in Table 1.

**Table 1:** Characteristics of studies included in the meta-analysis.

First Author	Year	Region	Tumor site	Genotyping method	GG	GC	GC	Total	HWE*	NOS
					case/control	case/control	case/control	case/control		
Miao	2016	Inland	Oral	Illumina	154/497	228/773	80/278	576/1552	0.468	7
Lin	2014	Inland	Laryngel	Taqman	31/139	110/220	63/81	204/440	0.71	7
Chu	2012	TaiWan	Oral	PCR-RFLP	54/54	242/196	174/175	470/425	0.94	7
Lung(1)	2013	HongKong	NP	PCR	24/497	88/1807	117/1472	229/3776	0.12	7
Lung(2)	2013	HongKong	NP	PCR	24/18	88/86	117/59	229/163	0.133	7
Huang	2014	Inland	NP	PCR-RFLP	23/36	73/110	64/54	160/200	0.154	7
Chen(1)	2016	TaiWan	Laryngel	Taqman	16/103	77/293	53/272	188/197	0.1	7
Chen(2)	2016	TaiWan	Oral	Taqman	71/103	241/293	200/272	658/668	0.12	7

HWE: Hardy-Weinberg equilibrium; PCR: polymerase chain reaction; PCR-RFLP: polymerase chain reaction-restriction fragment length polymorphism

*P value for HWE in control

NP: Nasopharygeal

### Quantitative synthesis

**Fig 2:**
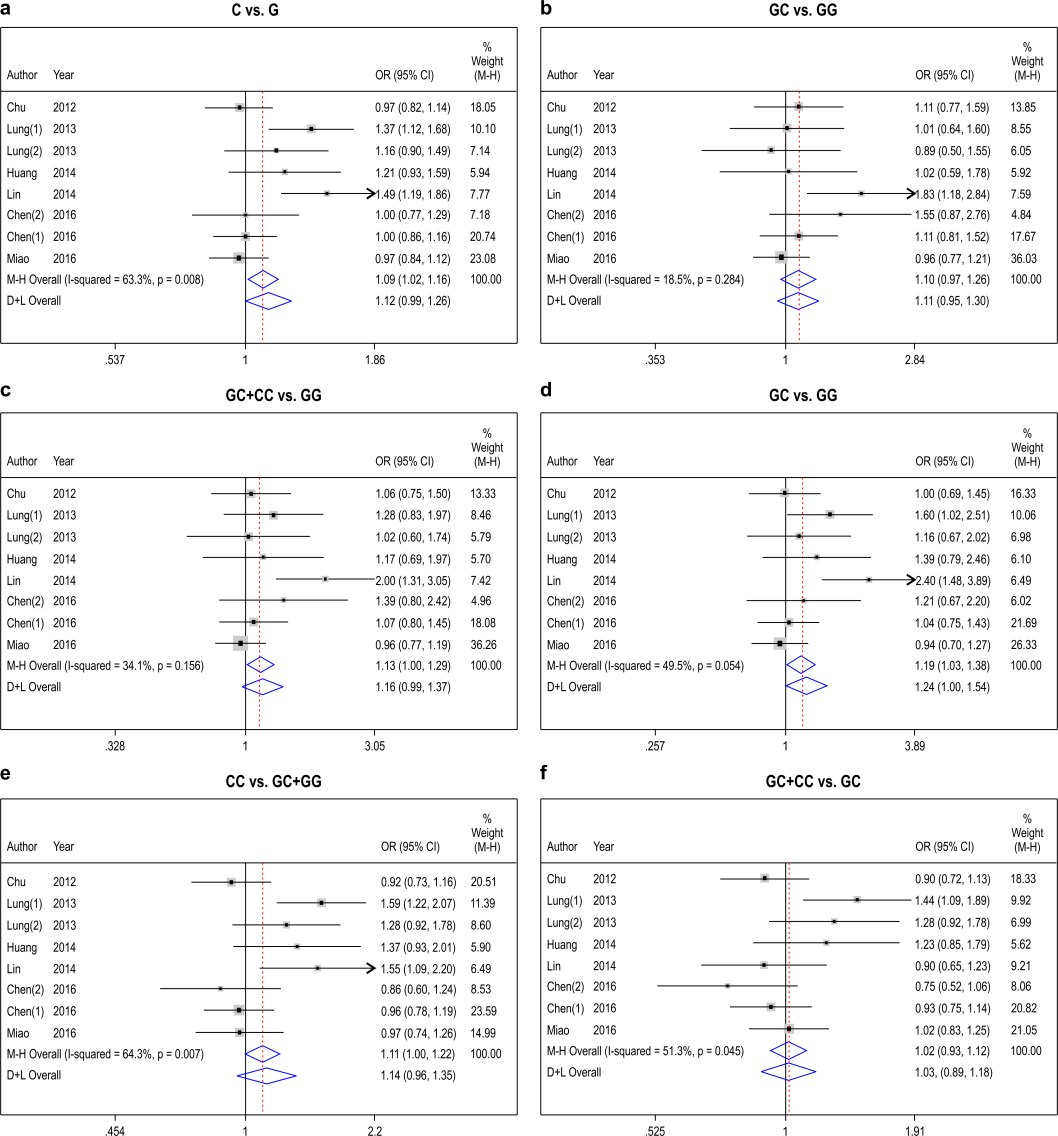
The forest of different model. (a): allelic contrast (b) heterozygote comparisons (c) dominant model (d) homozygote comparisons, (e) recessive models (f) additive models.

The ORs by a fixed-effect model (Mantel-Haenszel) were as follows: for the dominant model, GC+CC vs GG: OR = 1.13; 95%CI = 1.00∼1.29, p = 0.156 for heterogeneity, *I*^2^ = 34.1%; homozygote model, CC vs GG: OR = 1.19; 95%CI = 1.03∼1.38, p = 0.054 for heterogeneity, *I*^2^ = 49.5%; and heterozygote comparisons, GC vs. GG: OR = 1.10; 95%CI = 0.97∼1.26, p = 0.284 for heterogeneity, *I*^2^ = 18.5%. The ORs by a random-effect model (DerSimonian and Laird) were as follows: for the allelic contrast, C vs. G: OR = 1.12; 95%CI = 0.99∼1.26, p = 0.008 for heterogeneity, *I*^2^ = 63.3%; recessive models, CC vs. GC+GG: OR = 1.14; 95%CI = 0.96∼1.35, p = 0.007 for heterogeneity, *I*^2^ = 64.3%; and additive models, GG+CC vs GC: OR = 1.03; 95%CI = 0.89∼1.18, p = 0.045 for heterogeneity, *I*^2^ = 51.3% (Fig 2). These data revealed that the dominant model and homozygote model may be risk factors in Chinese patients for SSCHN development.

To assess the effect of other factors on the results, we performed subgroup analysis based on the tumor site, region, cancer type, and genotyping method.

In the subgroup analysis based on tumor site, the risks of cancer were significantly increased for laryngeal cancer (dominant model: OR = 1.76, 95%CI = 1.26∼2.46,P = 0.001; homozygote model: OR = 1.83, 95%CI = 1.25∼2.67, P = 0.002) and nasopharyngeal carcinoma (homozygote model: OR = 1.41, 95%CI = 1.05∼1.90, P = 0.022). In the subgroup analysis based on region, the ORs of the homozygote model are statistically significant for Hong Kong and Inland; significantly evaluated risk was not observed for Taiwan in these genetic models. The results in the subgroup analysis for PCR was significant (dominant model: OR = 1.42, 95%CI = 1.00∼2.01, P = 0.047; allelic contrast: OR = 1.28, 95%CI = 1.10∼1.50, P = 0.002) (Table 2).

**Table 2:** Main results of the pooled data in the meta-analysis.

	No. of studies	C vs. G			CC vs. GG			GC+CC vs. GG		
		OR(95% CI)	P	P^#^		OR(95% CI)	P	P^#^		OR(95% CI)	P	P^#^
Total	8	1.09(1.02–1.16)	0.008	0.015	1.19(1.03–1.38)	0.054	0.018	1.13(1.00–1.29)	0.156	0.05
Tumor site										
Oral	3	0.98(0.90–1.07)	0.956	0.655	0.99(0.82–1.20)	0.914	0.913	1.01(0.86–1.18)	0.793	0.914
Laryngeal	2	1.25(1.06–1.49)	0.023	0.009	*1*.*83(1*.*25–2*.*67)*	*0*.*082*	*0*.*002*	*1*.*76(1*.*26–2*.*46)*	*0*.*306*	*0*.*001*
NP	3	1.27(1.10–1.45)	0.569	0.001	1.41(1.05–1.90)	0.682	0.022	1.17(0.89–1.55)	0.82	0.266
Region										
HongKong	2	1.28(1.10–1.50)	0.315	0.002	*1*.*42(1*.*00–2*.*01)*	*0*.*382*	*0*.*047*	1.18(0.84–1.64)	0.528	0.343
Taiwan	3	1.00(0.89–1.09)	0.963	0.816	1.05(0.84–1.31)	0.86	0.69	1.11(0.90–1.37)	0.684	0.322
Inland	3	1.12(1.00–1.25)	0.005	0.046	*1*.*26(1*.*00–1*.*58)*	*0*.*005*	*0*.*049*	1.14(0.95–1.36)	0.009	0.152
Genotyping method										
Taqman	3	1.14(0.88–1.47)	0.011	0.082	1.43(0.84–2.43)	0.018	0.023	1.41(0.95–2.11)	0.060	0.008
PCR-RFLR	2	1.03(0.90–1.19)	0.166	0.672	1.10(0.81–1.51)	0.338	0.533	1.09(0.82–1.46)	0.756	0.558
PCR	2	1.28(1.10–1.50)	0.315	0.002	*1*.*42(1*.*00–2*.*01)*	*0*.*382*	*0*.*047*	1.17(0.84–1.64)	0.528	0.343
Illunima	1	0.97(0.84–1.12)	NA	0.690	0.94(0.70–1.27)	NA	0.018	0.96(0.77–1.19)	NA	0.696

NP: Nasoppharyngeal; P: p-value of the heterogenety; P#: p-value of the significance

### Sensitivity analysis and publication bias

**Fig 3:**
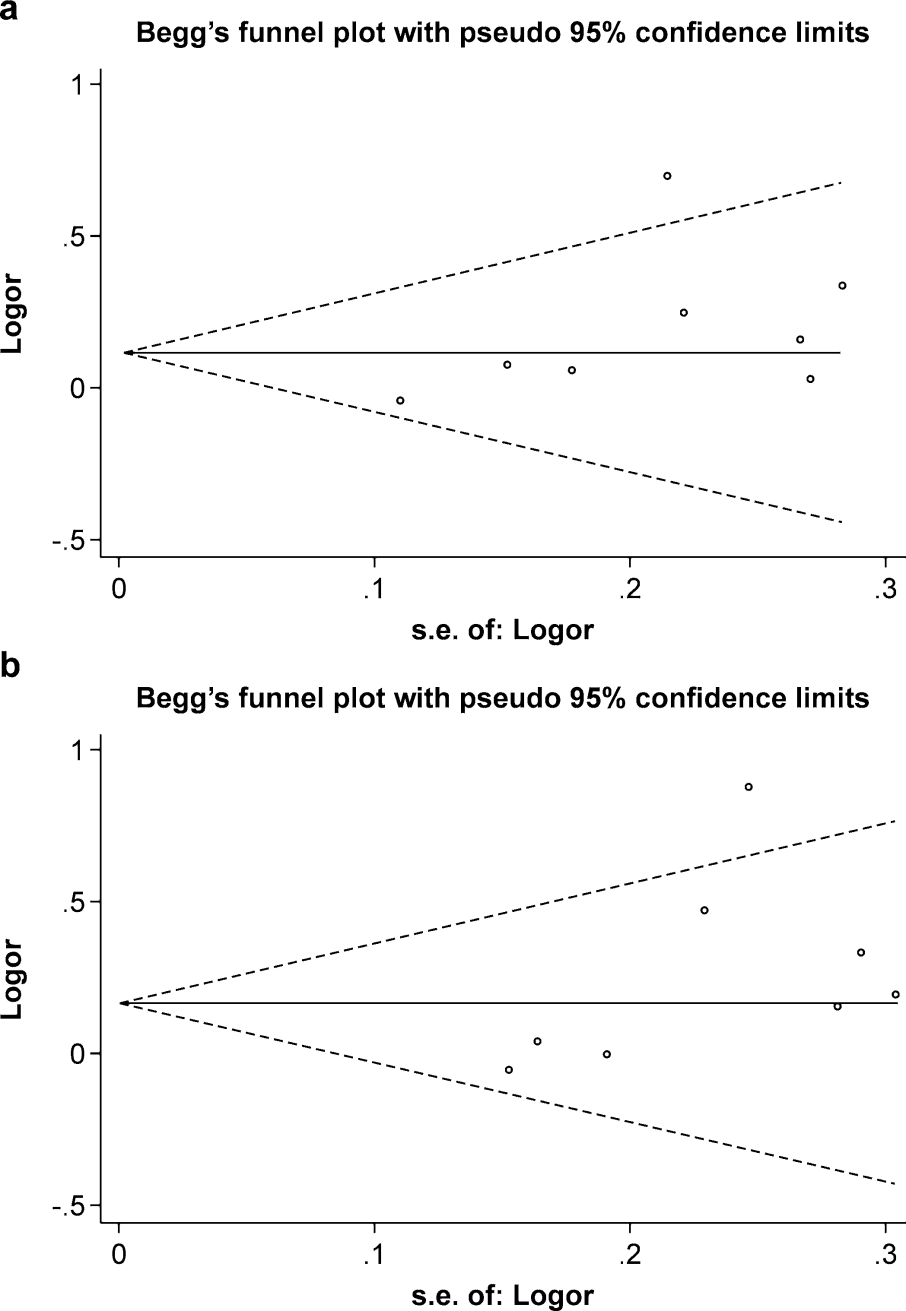
The funnel plot of different model. (a) dominant model; (b) homozygote model.

**Fig 4:**
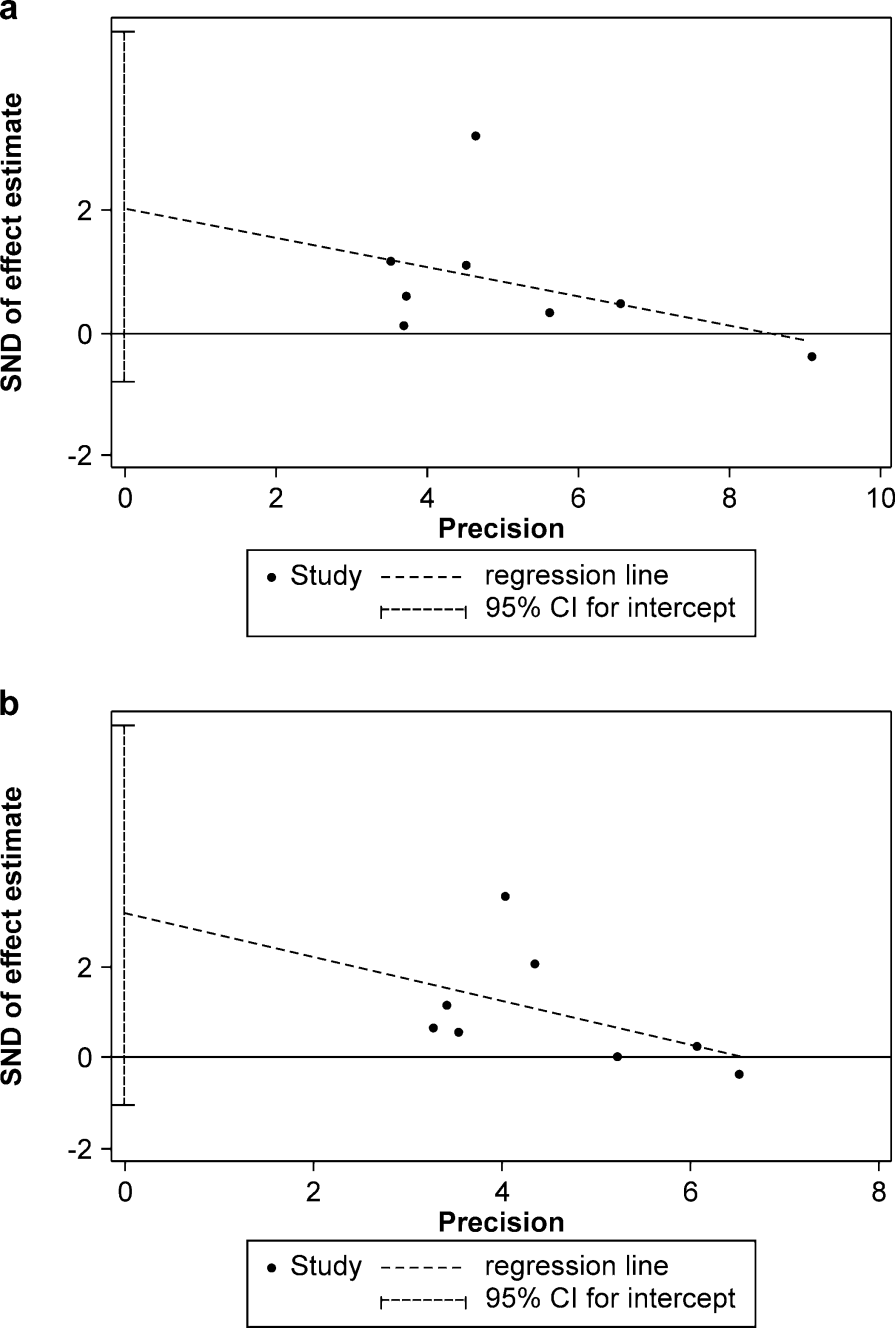
Egger`s publication bias plot of different model. (a) dominant model; (b) homozygote model.

Begg`s test was performed to determine the subjective assessment of the publication bias, and the symmetrical plots for the two genetic models were found (dominant model: z = 1.24, P = 0.216; homozygote model: z = 1.48, P = 0.138) (Fig 3); the Egger`s linear regression test also revealed that publication bias was not evident (dominant model: t = 1.76, P = 0.130; homozygote model: t = 1.84, P = 0.115) (Fig 4).

**Fig 5:**
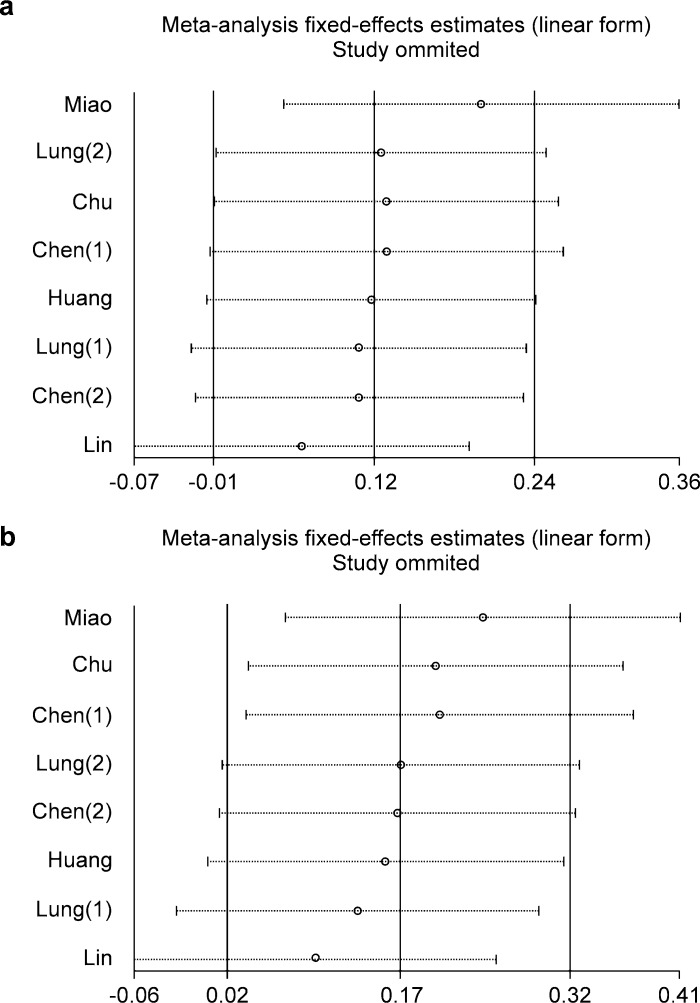
Sensitivity analysis to reflect the influence of the individual dataset to the pooled ORs in different model through deleting each study. (a) dominant model; (b) homozygote model.

Sensitivity analysis showed that the removal of individual documents in turn did not change the OR effect of the combined effect, which indicated that the results was stable in the meta-analysis (Fig 5).

## Discussion

MiRNA-146a polymorphisms may be associated with susceptibility to various tumors. Since 2008, when Jazdzewski et al. reported that the miRNA-146a heterozygous model had increased risk of thyroid cancer for the first time [30], there were a series of different findings related to SCCHN and the results of the meta-analyses were different. There are four meta-analyzes of head and neck cancer, of which 2 studies have synthesized thyroid cancer and head and neck cancer quantitatively[13, 14]; the other 2 studies[11, 12] contained research on SCCHN.

However, the SCCHN meta-analysis results were conflicting. One study performed by Xiang et al.[12] indicated the existence of the variant C allele of miR-146a correlated with increased risk of SCCHN, but no association could be observed in the Asian subgroup. The paper by Chen et al.[11] showed no significant differences in the association of the polymorphism of miR-146a and the SCCHN risk between patients and healthy controls, but the subgroup analysis results showed significant differences in this association in Chinese populations. Notably, a non-case-control study[22] was included by Chen et al.[11], and the heterogeneity of the results was large and no heterogeneity source was found. They did not take into account genetic differences between ethnicities and regions, and their results may not suitable for the Chinese population.

In this study, we found that the miRNA-146a polymorphisms were associated with SCCHN susceptibility, and that the dominant and homozygote models may have increased risk of SCCHN, but such did not emerge in heterozygote comparisons. The results were different from the study of Chen et al.[11], which may be related to the number of studies. Subgroup analysis found that this polymorphism also has a relationship with the tumor site and the region of the patients. The dominant model showed increased risk of laryngeal cancer and nasopharyngeal cancer, whereas the homozygote model may have an increased risk of laryngeal cancer. In the context of carcinogenesis, there are varying etiologic exposures associated with different cancers: in SCCHN, etiologic exposures may differ between individuals (within the tumor site) and are known to have different magnitudes of effect by tumor site[31]. Furniss et al.[32] revealed that *HPV16*-seropositive individuals have a higher risk of developing pharyngeal disease compared to other sites. Further, *HPV16* in SCCHN was associated with specific molecular alterations that may have broader implications for a cell`s transcriptome, and so, may differentially impact regulation by miRNAs. Genotyping by different methods showed obvious inconsistencies; the results indicated that the application of PCR may be more helpful in improving the accuracy of the trial.

The mechanism of how miRNA-146a polymorphisms increase the risk of HNC is unclear. The miRNA-146a polymorphism site is located in the precursor region of miRNA-146a, causing a mismatch of C: U in the stem-loop structure of the miRNA[33], which affects the expression of precursor miRNA146a to mature miRNA146[30]. The polymorphic loci significantly increased the breast cancer risk in Chinese Han women[34]; it also affects the development and prognosis of breast cancer[35]. These studies may help explain why the miRNA-146a polymorphism can increase the risk of SCCHN in Chinese patients.

However, there are deficiencies in this meta-analysis. Firstly, there is a slight heterogeneity of the results, which may be related to individual susceptibility, eating habits, and environmental factors. We have not been able to take them into account because of the small size of the dataset. Secondly, the diseases included in the studies have obvious regional characteristics. For example, the literatures of oral cancer mainly come from Taiwan, throat cancer mainly from the mainland, and the cases of the research by Lung et al.[25] were collected from the mainland and Hong Kong. Finally, the included studies had small sample sizes and there were a limited number of studies, which may reduce the statistical power of our results. In our studies, we didn’t explore whether the *HPV* have the effect on the results of miRNA-146a and the susceptibility to SCCHN, because the paper we contained in this study did not distinguish the *HPV*-positive SCCHN and *HPV*-negative SCCHN cases. Studies have revealed that the some genes patterns of allelic, chromosomal loss and the global gene expression profiles was different between the *HPV-*positive and *HPV*-negative SCCHN[36–38]. And one study has shown that the C-allele of *rs2910164* polymorphism in miRNA-146a was associated with the risk of nasopharyngeal cancer in Chinese[39]. Another study indicated that joint effect of *HPV16* seropositivity and each of these microRNA SNPs increased the risk of oral squamous cell carcinoma (OSCC), although no significant interaction was found for such joint effect on risk of OSCC[40]. According to these studies, we speculated that the *HPV* may have effect on the results of miRNA-146a and the susceptibility to SCCHN, and we will explore this problem in further studies.

In conclusion, this study show that the miRNA-146a polymorphism is associated with susceptibility to SCCHN in Chinese patients, and this polymorphism is related to aspects of the cancer type and the patient's region. In addition, this conclusion will need to be validated by a single disease case-control study with a large sample size.

## Supporting information

S1 File Search strategy. (PDF)

S1 Table Genetic-association-studies-checklist. (PDF)

S1 Fig PRISMA 2009 flow diagram. (PDF)
