# Supplementary material for: Association between a miRNA-146a polymorphism and susceptibility to head and neck squamous cell carcinoma in Chinese patients: A meta-analysis of 8 case–control studies
Source: PLoS One. 2017 Oct 19;12(10):e0186609. doi: 10.1371/journal.pone.0186609 (PMC5648221; doi:10.1371/journal.pone.0186609)
Supplement: S1 File — (PDF) [file pone.0186609.s001.pdf]

Title: Association between a microRNA-146a polymorphism and susceptibility to head and neck squamous cell carcinoma in Chinese patients: a meta-analysis of 8 case–control studies

Pubmed search strategy:

Search ((((((("Mouth Neoplasms"[Mesh]) OR (((((((((((((((((Mouth Neoplasm[Title/Abstract]) OR Neoplasm, Mouth[Title/Abstract]) OR Neoplasms, Oral[Title/Abstract]) OR Neoplasm, Oral[Title/Abstract]) OR Oral Neoplasm[Title/Abstract]) OR Oral Neoplasms[Title/Abstract]) OR Neoplasms, Mouth[Title/Abstract]) OR Cancer of Mouth[Title/Abstract]) OR Mouth Cancers[Title/Abstract]) OR Mouth Cancer[Title/Abstract]) OR Cancer, Mouth[Title/Abstract]) OR Cancers, Mouth[Title/Abstract]) OR Oral Cancer[Title/Abstract]) OR Cancer, Oral[Title/Abstract]) OR Cancers, Oral[Title/Abstract]) OR Oral Cancers[Title/Abstract]) OR Cancer of the Mouth[Title/Abstract]))) OR (("Laryngeal Neoplasms"[Mesh]) OR (((((((((((((((((Neoplasms, Laryngeal[Title/Abstract]) OR Laryngeal Neoplasm[Title/Abstract]) OR Neoplasm, Laryngeal[Title/Abstract]) OR Larynx Neoplasms[Title/Abstract]) OR Larynx Neoplasm[Title/Abstract]) OR Neoplasm, Larynx[Title/Abstract]) OR Neoplasms, Larynx[Title/Abstract]) OR Cancer of Larynx[Title/Abstract]) OR Larynx Cancers[Title/Abstract]) OR Laryngeal Cancer[Title/Abstract]) OR Cancer, Laryngeal[Title/Abstract]) OR Cancers, Laryngeal[Title/Abstract]) OR Laryngeal Cancers[Title/Abstract]) OR Larynx Cancer[Title/Abstract]) OR Cancer, Larynx[Title/Abstract]) OR Cancers, Larynx[Title/Abstract]) OR Cancer of the Larynx[Title/Abstract]))) OR (("Nasopharyngeal Neoplasms"[Mesh]) OR (((((((((((((((((Nasopharyngeal Neoplasm[Title/Abstract]) OR Neoplasm, Nasopharyngeal[Title/Abstract]) OR Neoplasms, Nasopharyngeal[Title/Abstract]) OR Nasopharynx Neoplasms[Title/Abstract]) OR Nasopharynx Neoplasm[Title/Abstract]) OR Neoplasm, Nasopharynx[Title/Abstract]) OR Neoplasms, Nasopharynx[Title/Abstract]) OR Cancer of Nasopharynx[Title/Abstract]) OR Nasopharynx Cancers[Title/Abstract]) OR Nasopharyngeal Cancer[Title/Abstract]) OR Cancer, Nasopharyngeal[Title/Abstract]) OR Cancers, Nasopharyngeal[Title/Abstract]) OR Nasopharyngeal Cancers[Title/Abstract]) OR Nasopharynx Cancer[Title/Abstract]) OR Cancer, Nasopharynx[Title/Abstract]) OR Cancers, Nasopharynx[Title/Abstract]) OR Cancer of the Nasopharynx[Title/Abstract]))) OR ((("Head and Neck Neoplasms"[Mesh])) OR (((((((((((((((((((((((head[Title/Abstract] AND neck neoplasms[Title/Abstract])) OR (Neoplasms, Head[Title/Abstract] AND Neck[Title/Abstract])) OR Head, Neck Neoplasms[Title/Abstract]) OR (Cancer of Head[Title/Abstract] AND Neck[Title/Abstract])) OR (Head[Title/Abstract] AND Neck Cancer[Title/Abstract])) OR (Cancer of the Head[Title/Abstract] AND Neck[Title/Abstract])) OR Upper Aerodigestive Tract Neoplasms[Title/Abstract]) OR UADT Neoplasms[Title/Abstract]) OR Neoplasm, UADT[Title/Abstract]) OR Neoplasms, UADT[Title/Abstract]) OR UADT Neoplasm[Title/Abstract]) OR Neoplasms, Upper

Aerodigestive Tract[Title/Abstract]) OR Head Neoplasms[Title/Abstract]) OR Neoplasms, Head[Title/Abstract]) OR Neck Neoplasms[Title/Abstract]) OR Neoplasms, Neck[Title/Abstract]) OR Cancer of Head[Title/Abstract]) OR Head Cancer[Title/Abstract]) OR Cancer of the Head[Title/Abstract]) OR Cancer of Neck[Title/Abstract]) OR Neck Cancer[Title/Abstract]) OR Cancer of the Neck[Title/Abstract])))) AND (("MIRN146 microRNA, human" [Supplementary Concept]) OR (((((((((microRNA-146, human[Title/Abstract]) OR miR-146, human[Title/Abstract]) OR hsa-mir-146[Title/Abstract]) OR MIRN146b microRNA, human[Title/Abstract]) OR hsa-mir-146a-5p, human[Title/Abstract]) OR MIRN146A microRNA, human[Title/Abstract]) OR microRNA 146a, human[Title/Abstract]) OR hsa-mir-146a microRNA[Title/Abstract]) OR miR-146a, human[Title/Abstract])).

Embase search strategy:

'head and neck cancer'/exp OR 'neoplasms, head and neck':ab,ti OR 'head, neck neoplasms':ab,ti OR 'cancer of head and neck':ab,ti OR 'head and neck cancer':ab,ti OR 'cancer of the head and neck':ab,ti OR 'upper aerodigestive tract neoplasms':ab,ti OR 'uadt neoplasms':ab,ti OR 'neoplasm, uadt':ab,ti OR 'neoplasms, uadt':ab,ti OR 'uadt neoplasm':ab,ti OR 'neoplasms, upper aerodigestive tract':ab,ti OR 'head neoplasms':ab,ti OR 'neoplasms, head':ab,ti OR 'neck neoplasms':ab,ti OR 'neoplasms, neck':ab,ti OR 'cancer of head':ab,ti OR 'head cancer':ab,ti OR 'cancer of the head':ab,ti OR 'cancer of neck':ab,ti OR 'neck cancer':ab,ti OR 'cancer of the neck':ab,ti OR 'nasopharynx cancer'/exp OR 'nasopharyngeal neoplasm':ab,ti OR 'neoplasm, nasopharyngeal':ab,ti OR 'neoplasms':ab,ti OR 'nasopharyngeal':ab,ti OR 'nasopharynx neoplasms':ab,ti OR 'nasopharynx neoplasm':ab,ti OR 'neoplasm, nasopharynx':ab,ti OR 'neoplasms, nasopharynx':ab,ti OR 'cancer of nasopharynx':ab,ti OR 'nasopharynx cancers':ab,ti OR 'nasopharyngeal cancer':ab,ti OR 'cancer, nasopharyngeal':ab,ti OR 'cancers, nasopharyngeal':ab,ti OR 'nasopharyngeal cancers':ab,ti OR 'nasopharynx cancer':ab,ti OR 'cancer, nasopharynx':ab,ti OR 'cancers, nasopharynx':ab,ti OR 'cancer of the nasopharynx':ab,ti OR 'larynx tumor'/exp OR 'neoplasms, laryngeal':ab,ti OR 'laryngeal neoplasm':ab,ti OR 'neoplasm,laryngeal':ab,ti OR 'larynx neoplasms':ab,ti OR 'larynx neoplasm':ab,ti OR 'neoplasm, larynx':ab,ti OR 'neoplasms, larynx':ab,ti OR 'cancer of larynx':ab,ti OR 'larynx cancers':ab,ti OR 'laryngeal cancer':ab,ti OR 'cancer, laryngeal':ab,ti OR 'cancers, laryngeal':ab,ti OR 'laryngeal cancers':ab,ti OR 'larynx cancer':ab,ti OR 'cancer, larynx':ab,ti OR 'cancers, larynx':ab,ti OR 'cancer of the larynx':ab,ti OR 'mouth tumor'/exp OR 'mouth neoplasm':ab,ti OR 'neoplasm, mouth':ab,ti OR 'neoplasms, oral':ab,ti OR 'neoplasm, oral':ab,ti OR 'oral neoplasm':ab,ti OR 'oral neoplasms':ab,ti OR 'neoplasms, mouth':ab,ti OR 'cancer of mouth':ab,ti OR 'mouth cancers':ab,ti OR 'mouth cancer':ab,ti OR 'cancer, mouth':ab,ti OR 'cancers, mouth':ab,ti OR 'oral cancer':ab,ti OR 'cancer, oral':ab,ti OR 'cancers, oral':ab,ti OR 'oral cancers':ab,ti OR 'cancer of the mouth':ab,ti AND ('microrna 146a'/exp OR 'microrna-146, human':ab,ti OR 'mir-146, human':ab,ti OR 'hsa-mir-146':ab,ti OR 'mirn146b microrna, human':ab,ti OR 'hsa-mir-146a-5p,

human':ab,ti OR 'mirn146a microrna, human':ab,ti OR 'microrna 146a, human':ab,ti OR 'hsa-mir-146a microrna':ab,ti OR 'mir-146a, human':ab,ti)

Web of science search strategy:

#1

TIS =(Neoplasm*, Head and Neck OR Head, Neck Neoplasm* OR Cancer of Head and Neck OR Head and Neck Cancer OR Cancer of the Head and Neck OR Upper Aerodigestive Tract Neoplasm* OR UADT Neoplasm* OR Neoplasm, UADT OR Neoplasm*, UADT OR UADT Neoplasm OR Neoplasm*, Upper Aerodigestive Tract OR Head Neoplasm* OR Neoplasm*, Head OR Neck Neoplasm* OR Neoplasm*, Neck OR Cancer of Head OR Head Cancer OR Cancer of the Head OR Cancer of Neck OR Neck Cancer OR Cancer of the Neck)

#2

TIS =(Nasopharyngeal Neoplasm OR Neoplasm, Nasopharyngeal OR Neoplasm* OR Nasopharyngeal OR Nasopharynx Neoplasm* OR Nasopharynx Neoplasm OR Neoplasm, Nasopharynx OR Neoplasm*, Nasopharynx OR Cancer of Nasopharynx OR Nasopharynx Cancer* OR Nasopharyngeal Cancer OR Cancer, Nasopharyngeal OR Cancer*, Nasopharyngeal OR Nasopharyngeal Cancer* OR Nasopharynx Cancer OR Cancer, Nasopharynx OR Cancer*, Nasopharynx OR Cancer of the Nasopharynx)

#3

TIS=(Neoplasm*, Laryngeal OR Laryngeal Neoplasm OR Neoplasm,Laryngeal OR Larynx Neoplasm* OR Larynx Neoplasm OR Neoplasm, Larynx OR Neoplasm*, Larynx OR Cancer of Larynx OR Larynx Cancer* OR Laryngeal Cancer OR Cancer, Laryngeal OR Cancer*, Laryngeal OR Laryngeal Cancer* OR Larynx Cancer OR Cancer, Larynx OR Cancer*, Larynx OR Cancer of the Larynx)

#4

TIS=(Mouth Neoplasm OR Neoplasm, Mouth OR Neoplasm*, Oral OR Neoplasm, Oral OR Oral Neoplasm OR Oral Neoplasm* OR Neoplasm*, Mouth OR Cancer of Mouth OR Mouth Cancer* OR Mouth Cancer OR Cancer, Mouth OR Cancer*, Mouth OR Oral Cancer OR Cancer, Oral OR Cancer*, Oral OR Oral Cancer* OR Cancer of the Mouth)

#5

TIS=(microRNA-146, human OR miR-146, human OR hsa-mir-146 OR MIRN146b microRNA, human OR hsa-mir-146a-5p, human OR MIRN146A microRNA, human OR microRNA 146a, human OR hsa-mir-146a microRNA OR miR-146a, human)

#6: #1AND#2AND#3AND#4AND#5

CNKI database:

#1: 头颈肿瘤 或并 鼻咽癌 或并 口腔癌 或并 喉癌 或并 下咽癌

#2 microRNA-14a 或并 miRNA-146a 或并 MiR-146a 或并 miR-14a 或并 rs2910164

#3 基因多态性 或并 单核苷酸多态性 或并多态性

Wangfang databse:

“头颈肿瘤 ＋ 鼻咽癌 + 口腔癌 + 喉癌 + 下咽癌‘’ 与“microRNA-14a + miRNA-146a + MiR-146a + miR-14a + rs2910164” 与 “基因多态性 + 单核苷酸多态性 + 多态性”
