# Supplementary material for: Association between a miRNA-146a polymorphism and susceptibility to head and neck squamous cell carcinoma in Chinese patients: A meta-analysis of 8 case–control studies
Source: PLoS One. 2017 Oct 19;12(10):e0186609. doi: 10.1371/journal.pone.0186609 (PMC5648221; doi:10.1371/journal.pone.0186609)
Supplement: S1 Table — (PDF) [file pone.0186609.s002.pdf]

Meta-analysis on Genetic Association Studies Checklist | PLOS ONE

	Item	Section name and paragraph number within manuscript
	**Introduction**	
1	Provide a detailed justification for the polymorphism studied; if a single polymorphism was analyzed, give details as to why others were not included in the meta-analysis.	Introduction:3
2	Provide a detailed justification for the population(s) and clinical condition studied.	Introduction：1−3
	**Methods**	
3	Provide full details of the search strategy employed; outline the full electronic search strategy –specific combination of keywords and any limits applied- for at least one database. Specify whether synonyms of polymorphisms/genes (e.g. SNP number) were searched.	Publication Search:4
4	Report full details on the inclusion and exclusion criteria applied for selecting studies. *Please list the excluded articles and the reasons for exclusion of each article in a supplementary file.*	PRISMA 2009 flow diagram
5	Provide details on how the quality of the studies included in the analyses was assessed.	Data Extraction:5
6	Describe steps taken to contact study authors to identify additional studies and to request missing data.	
7	Describe how environmental effects were adjusted for, if this adjustment was not conducted, outline the reasons for this.	Statistical Analysis:6
8	Describe the methods of handling heterogeneity/between-study variance.	Statistical Analysis:8、10
9	Describe how the Hardy-Weinberg equilibrium and linkage disequilibrium were assessed.	Statistical Analysis:6
10	Describe and justify the choice of model for the analyses (per-allele vs per-genotype vs genetic model-free, random effects vs fixed effects).	Statistical Analysis:8
11	Describe whether a sensitivity analysis has been completed.	Statistical Analysis:9

12	Describe whether an assessment of the effects of population stratification has been conducted.	Statistical Analysis:7
13	Describe whether study-specific results have been assessed and if so the reasons for this (e.g. forest plot).	Statistical Analysis:7
	**Results**	
14	Include flow diagram for the studies included in the meta-analysis as the first figure for the manuscript	Study Identification:11
15	Report details on allele/genotype prevalence.	Study Identification:12
16	Report the effect size estimates and p values for each analysis.	Quantitative Synthesis:13
	**Discussion**	
17	Discuss the limitations of the meta-analysis, including genotyping errors/bias and publication bias.	Discussion:22
18	If the meta-analysis identifies an association within a subgroup of the population studied but not another, discuss the implications of these results, and if applicable the possibility of subgroup-specific publication bias.	Discussion:20
19	Discuss the suitability of the sample size employed to the research question and the power of the study.	Discussion:23
