# Supplementary material for: Association between a miRNA-146a polymorphism and susceptibility to head and neck squamous cell carcinoma in Chinese patients: A meta-analysis of 8 case–control studies
Source: PLoS One. 2017 Oct 19;12(10):e0186609. doi: 10.1371/journal.pone.0186609 (PMC5648221; doi:10.1371/journal.pone.0186609)
Supplement: S1 Fig — (PDF) [file pone.0186609.s003.pdf]

PRISMA 2009 Flow Diagram

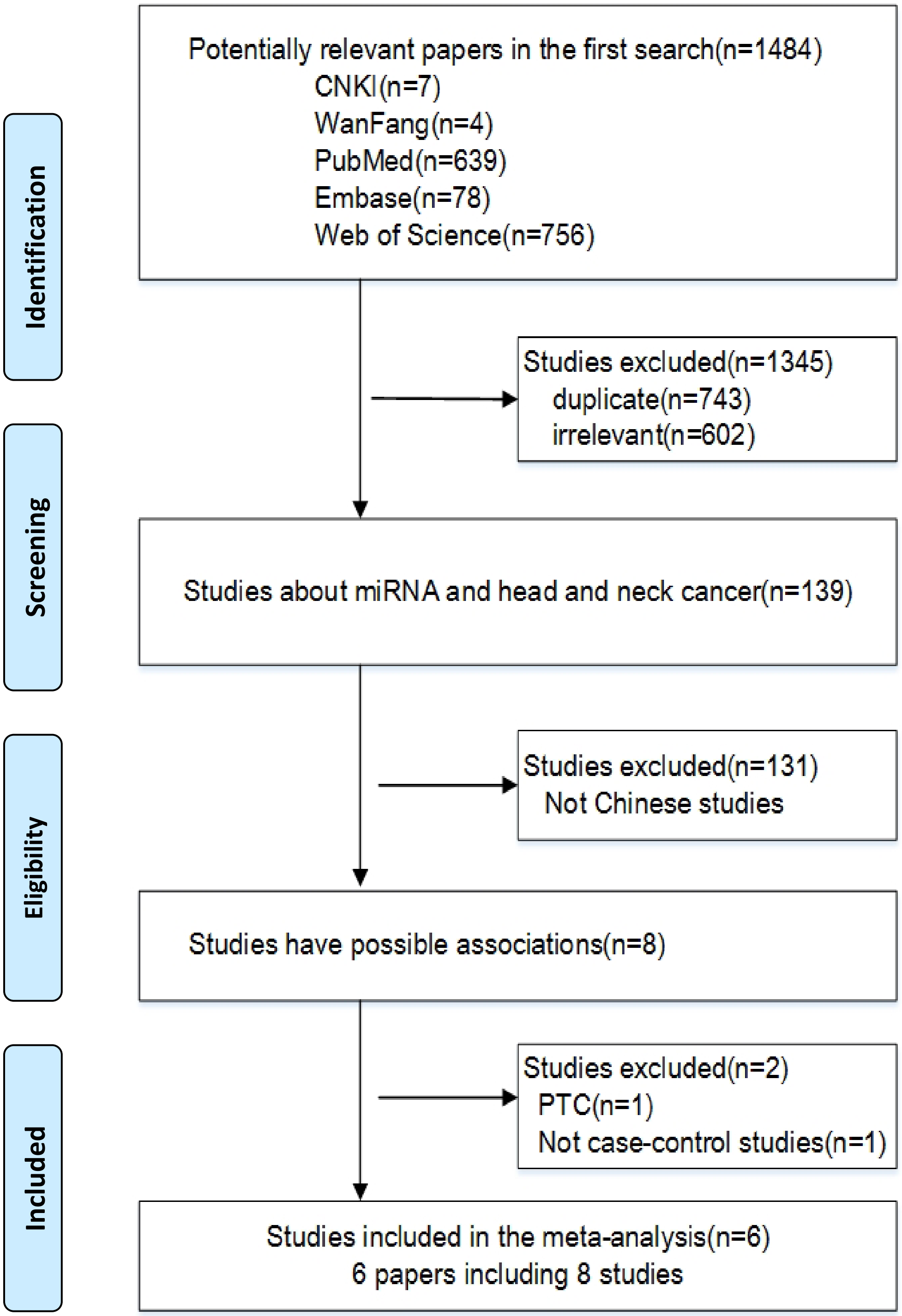

From: Moher D, Liberati A, Tetzlaff J, Altman DG, The PRISMA Group (2009). *P*referred *R*eporting *I*tems for *S*ystematic Reviews and *M*eta-*A*nalyses: The PRISMA Statement. PLoS Med 6(7): e1000097. doi:10.1371/journal.pmed1000097

For more information, visit www.prisma-statement.org.
